# Protein kinase C and endothelial dysfunction in select vascular diseases

**DOI:** 10.3389/fcvm.2025.1618343

**Published:** 2025-08-25

**Authors:** Shawn Kant, Jun Feng

**Affiliations:** ^1^ Cardiothoracic Surgery Research Laboratory, Department of Medicine, Rhode Island Hospital, Brown University Health, Providence, RI, United States; ^2^ Department of Surgery, Heart Institute, Morsani College of Medicine, University of South Florida, Tampa, FL, United States

**Keywords:** PKC, endothelial cell, vascular function, diabetes, hypertension, ischemia-reperfusion

## Abstract

Protein kinases have crucial roles in intracellular signal transduction pathways that affect a wide range of biochemical processes, including apoptosis, metabolism, proliferation, and protein synthesis. Vascular endothelial cells are important regulators of vasomotor tone, tissue/organ perfusion, and inflammation. Since its discovery in the late 1970s, a growing body of literature implicates protein kinase C (PKC) in pathways involving angiogenesis, endothelial permeability, microvascular tone, and endothelial activation. Hence the objective of this review, to characterize the role of PKC in vascular endothelial cells. After discussing the basic principles of PKC structure and function, the focus shifts to abnormal PKC activity driving endothelial dysfunction in three major pathologies whose hallmark is significant vascular disease: diabetes mellitus, hypoxia/ischemia-reperfusion injury, and hypertension. Themes addressed include endothelial cell cycle derangement, endothelial oxidative stress, endothelial activation/inflammation, and impaired endothelial barrier integrity. Achieving a comprehensive understanding of endothelial cell-PKC pathophysiology may lead to development of new therapeutic targets for mitigating morbidity and mortality in these disease states.

## 1 Introduction

Consisting of over 500 proteins, and about 2% of the human genome, protein kinases are one of the largest and most diverse families of proteins in the human body. Kinases catalyze phosphorylation, a form of post-translational modification that is critical for intracellular signal transduction pathways involving enzymes, transmembrane receptors, and other proteins. Indeed, up to two thirds of intracellular proteins may be phosphorylated at any given time, with phosphorylation often acting as an on/off switch for a large set of pathways involved in processes including metabolism, proliferation, apoptosis, and protein synthesis (1). Due to their near-ubiquitous presence across diverse biochemical pathways that influence all domains of physiology and pathophysiology, protein kinases have routinely been cited as potential therapeutic targets for drug development in clinical research.

Residing at the blood-tissue interface of every organ system in the body, the vascular endothelium plays a critical role in multiple important physiologic processes. Generally speaking, vascular endothelial cells secrete endothelium-derived vasoconstrictors (e.g., endothelin-1 and thromboxane A2) and vasodilators (nitric oxide, prostacyclin, endothelium-derived hyperpolarizing factor) whose balance and interplay underpins vasomotor tone (2). This in turn regulates blood flow, and subsequently oxygen delivery to different tissues and organs. Moreover, vascular endothelial cells form an important barrier through which other cells and molecules must pass through from the systemic circulation to reach various organ systems, with activation of vascular endothelial cells triggering enhanced permeability, coagulation, and tissue extravasation. In specific tissue beds, vascular endothelial cells may serve more specific functions, such as glomerular endothelial cells' role in renal fluid filtration. Endothelial dysfunction, observed in numerous diseases characterized by metabolic dysregulation, deranges all of these critical functions, and is increasingly recognized as an important mediator of observed pathology.

Protein kinase C (PKC) was first discovered in 1977, as a cyclic nucleotide independent, calcium and lipid dependent kinase purified from rat and bovine cerebellum (3, 4). Since then, a flurry of research has implicated PKC isoforms as major regulators of a diverse array of biochemical functions, including cell cycle regulation, tumorigenicity, differentiation, and inflammation. With respect to vascular endothelial cells, this translates into PKC participating in pathways involving angiogenesis, endothelial permeability, microvascular tone, and endothelial activation. This review first discusses basic principles of PKC structure, function, and endothelial localization before moving into a detailed discussion of links between abnormal PKC activity and endothelial dysfunction in three major disease states: diabetes mellitus, hypertension, and ischemia-reperfusion.

## 2 Structure, endothelial localization, and function of PKC

### 2.1 Isoforms and key structural features

All members of the PKC family share certain conserved features, including a catalytic domain composed of ATP/substrate-binding and catalytic motifs, and a regulatory domain that maintains the enzyme in an inactive conformation when not needed (5). The PKC regulatory domain, which resides at the amino terminal of the protein, consists of an autoinhibitory pseudo substrate domain (with alanine replacing the serine/threonine phosphoacceptor site), and two membrane targeting modules C1 and C2 (5).

PKC isoforms are divided into three major subfamilies based on differences in amino-terminal regulatory domain structure (5). First, there are the conventional isoforms: PKCα, β1, and β2, and γ. Each contains four conserved (C1–C4) and five variable (V1–V5) regions (6). The C1 domain acts as a diacylglycerol (DAG)/phorbol ester (PMA) binding center with cysteine-rich zinc finger-like motifs (5, 6). Conventional PKC regulatory domains contain a C2 domain capable of calcium-dependent binding to anionic phospholipids (5, 7). Next, there are the novel PKCs, which can be subdivided into the related δ/θ and ε/η variants. These isoforms also contain twin C1 domains and a C2 domain, although arranged in a different order compared to the linear sequence of conventional PKC isoforms, and lack the calcium-coordinating acidic C2 domain residues found in conventional isoforms (5, 8).

As a consequence, novel PKC isoforms can be strongly activated by DAG and PMA alone, as opposed to requiring calcium unlike conventional PKC isoforms. Finally, there are the atypical PKCs, consisting of PKCζ, ι, and λ. Like novel PKCs, atypical PKCs also lack a calcium-sensitive C2 domain (5, 9). However, unlike novel PKCs, the C1 domain of atypicals consists of only one cysteine-rich membrane targeting structure that can bind PIP3 or ceramide, and also contains a protein-protein interaction region that can interact with various PB1 containing scaffolding proteins (10, 11).

### 2.2 Endothelial localization of PKC

In general, extensive evidence exists supporting the notion that many isoforms of PKC are expressed in endothelial cells, including PKCα, β, and ε (12–15). Regarding other, less well studied isoforms, the data is more conflicting. Mattila et al. did not detect PKCγ or δ expression in human umbilical vein endothelial cells (15). While Mattila et al. did detect PKCζ in human coronary artery endothelial cells, Staiger et al. did not (15, 16). Meanwhile, Tang et al. found that rat capillary endothelial cells expressed PKCθ, which they implicated in cell cycle progression, endothelial mitosis, and maintenance of normal actin cytoskeletal structure (17). Staiger et al. determined that PKCι is present in human coronary artery endothelial cells, and mediates lipid-induced apoptosis in atherosclerotic lesions (16). Discrepancies in the literature, such as expression of certain PKC isoforms in some endothelial cell models but not others, raises the possibility that endothelial cells in different vascular beds may differentially express the various isoforms of PKC; this is a matter worthy of future investigation to better resolve conflicting data.

### 2.3 PKC pathways

**Figure 1:**
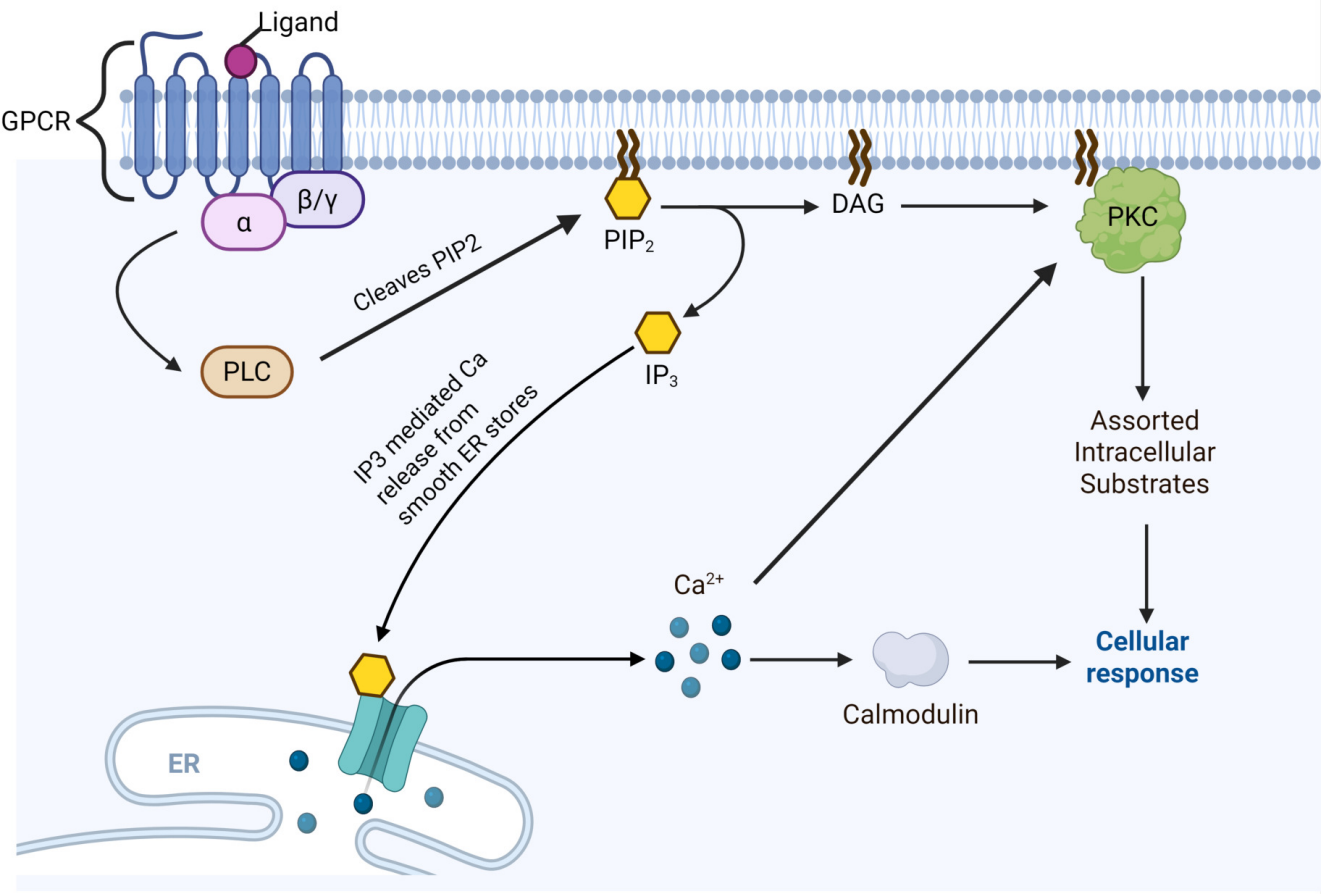
PKC activation pathway. Note that straight arrowheads indicate activation and blunt arrows indicate inhibitory activity. Typically begins with ligand-based activation of a G-protein Coupled Receptor (GPCR). This is followed by alpha subunit dissociation from the GPCR and activation of phospholipase C (PLC), which in turn cleaves PIP2 into IP3 and diacylglycerol (DAG). IP3 mediates calcium release from smooth ER stores. DAG directly activates membrane-bound protein kinase C (PKC), which activates an assortment of intracellular substrates via phosphorylation. Calcium may directly co-activate PKC, or enhance PKC-mediated effects via calcium-binding proteins such as calmodulin. Created in BioRender. Kant, S. (2025) https://BioRender.com/rovtois.

Most pathways of PKC activation begin with engagement of a growth factor or cytokine receptor, which in turn activate membrane bound phospholipase C (PLC). PLC then cleaves membrane-bound PIP2 to IP3 and DAG (Figure 1). IP3 diffuses into the cytosol and opens IP3-gated calcium channels on smooth ER stores, releasing calcium into the intracellular space. DAG proceeds along the membrane to, either alone or in conjunction with calcium, activate membrane-bound PKC, which then proceeds to phosphorylate serine/threonine residues on a vast array of potential substrates. Furthermore, DAG accumulation has also been shown to rapidly translocate conventional PKC isoforms from intracellular stores to the plasma membrane, often through interactions with RACK proteins (5).

Importantly, PKC activation does not require direct phosphorylation. In fact, studies have shown that the appropriate phosphorylation required for catalytic competence occurs soon after the enzyme is synthesized; therefore, most PKC isoforms are constitutively phosphorylated at their catalytic sites (18). Instead, membrane translocation of PKC remains the most reliable way to assess PKC activation (18). For conventional PKCs in particular, the actions of which have been most extensively studied, engagement of plasma membrane phosphatidylserine and DAG with the C1/C2 domains triggers high-affinity binding and a conformational change that frees the autoinhibitory pseudosubstrate domain from the substrate-binding pocket, therein allowing PKC to begin phosphorylating target substrates (5). Conversely, DAG metabolism can promote release of PKCs from the membrane.

DAG and calcium are the major activators of PKC, and anything that promotes DAG production and/or elevated intracellular calcium concentrations will act to increase PKC activity. Moreover, pharmacologic agents that mimic effects of DAG such as phorbol esters can also stimulate PKC activation (18). Meanwhile, PI3 kinase and PDK-1 have both been shown to activate PKC ζ through phosphorylation of threonine 410 in the PKCζ activation loop (19). Curiously, efforts to find endogenous direct inhibitors of PKC have yielded little results, with one of the only notable studies being that by Balazovich et al., who reported purification of an endogenous PKC inhibitor PKC-I from human neutrophils (20).

Finally, we present a brief overview of PKC targets in endothelial cells specifically, which will be explored in greater detail in subsequent sections on disease states. First, the conventional PKC isoforms are potent histone kinases (21). PKC also targets Gi, the inhibitory GTP binding protein. Phosphorylation of Gi activates adenylyl cyclase through facilitating dissociation of the alpha-1 subunit from the inactive enzyme (21). Various PKC isoforms promote angiogenic activity in human endothelial cells through induction of vascular endothelial growth factor (VEGF), phosphorylation of extracellular signal-related kinase (ERK) and protein kinase D (12, 22). Next, PKC isoforms target cell junction proteins, cadherins, and transient receptor potential canonical-1 (TRPC-1) channels to affect endothelial barrier function (23). Furthermore, PKC isoforms modulate atherogenesis and inflammation through stimulation of inflammatory cytokine activity, inhibition of anti-inflammatory mediators such as antithrombin, and enhanced leukocyte-endothelial cell adhesion in the microvasculature (24, 25).

## 3 PKC and endothelial dysfunction in diabetes mellitus

### 3.1 Background

Diabetes mellitus continues to grow in prevalence around the world. Per World Health Organization metrics, over 400 million adults globally suffer from either Type 1 or Type 2 diabetes, driven chiefly by ever-increasing incidence of type 2 diabetes (T2DM) in conjunction with the obesity epidemic ravaging large swaths of the developed world (26). Most concerningly, diagnosis of T2DM in children and adolescents under the age of 20 seems to be growing year after year. One study from 2021 identifies China, India, and the United States as sources of the highest estimated number of incident cases (27). A growing diabetic population worldwide will likely increase the incidence of diabetes-specific complications, chiefly diabetic kidney disease, peripheral arterial disease, retinopathy, and other macro and microvascular complications often seen in patients with longstanding diabetes (28).

Cardiovascular disease has long been identified as a leading cause of morbidity and mortality among patients with T2DM (29). Pathological vascular remodeling, including accelerated atherosclerosis, hypertrophic remodeling, reduced lumen diameter, and impaired vasomotor and myogenic tone have all been extensively characterized in diabetic vasculature (29–31). The end result is impaired organ perfusion across a variety of tissue vascular beds. Furthermore, growing evidence suggests that microvascular complications generally predate onset of macrovascular disease, and microvascular complications are associated with higher risk of macrovascular disease (32).

The last two decades have seen a flurry of research attempting to better characterize the mechanisms driving hyperglycemia-induced microvascular dysfunction, and it has become clear that these mechanisms are incredibly multifactorial in nature. Here, we explore the role of PKC in some of these pathways, beginning with studies implicating PKC in generalized systemic endothelial dysfunction in diabetic microvessels, before specifically addressing the impact of PKC on two critical microvascular complications of DM: diabetic nephropathy and diabetic retinopathy.

### 3.2 General observations

Hyperglycemic/diabetic phenotypes have been shown to induce expression of specific isoforms of PKC in specific vascular beds. Beginning with animal models, a streptozotocin (STZ)-induced mouse model of diabetes (STZ-DM) showed increased PKCα immunoreactivity in renal glomeruli and interstitial capillaries, cardiac capillaries, skeletal muscle capillaries, and large artery endothelial cells (33). The authors of the aforementioned study did not detect increased (or decreased) immunoreactivity of other PKC isoforms in those same tissues. Meanwhile, in another STZ-DM mouse model, diabetic mouse aortic endothelial cells exhibited increased cell membrane PKCβ2 activity, along with increased total DAG content without significant changes in PKCα levels (34). Of note, in a separate study, the same authors noted that chronic high glucose exposure increased DAG levels and PKCβ protein in cultured bovine and rat aortic endothelial cells (35). In addition, Inoguchi et al. observed increased phosphorylation of MARCKS, an intracellular substrate of PKC, providing further evidence for increased PKC activity in endothelial cells exposed to hyperglycemic states (34, 35).

Turning to human studies, human coronary arterioles and coronary artery endothelial cells taken from diabetic patients prior to undergoing cardiac surgery noted significantly increased PKC protein expression, in conjunction with increased levels of NADH, increased NADH/NAD+ ratios, and impaired coronary arteriolar endothelium-dependent vasodilatory responses (36). Microvessels taken from human skeletal muscle tissue displayed similar characteristics, insofar as diabetic patients exhibited decreased baseline vasomotor responses to the endothelium dependent vasodilators ADP and substance P relative to controls (37). The severity of hyperglycemia also appeared to play a role in the severity of microvascular dysfunction, as microvessels from patients with uncontrolled diabetes (average A1c of 9) had significantly more diminished responses to ADP and substance P than microvessels from patients with controlled diabetes (average A1c of 6.3) (37). As for PKC, diabetic skeletal microvessels exhibited significantly higher PKCα and β levels than their nondiabetic counterparts (37).

### 3.3 PKC and endothelial nitric oxide metabolism in diabetes

**Figure 2:**
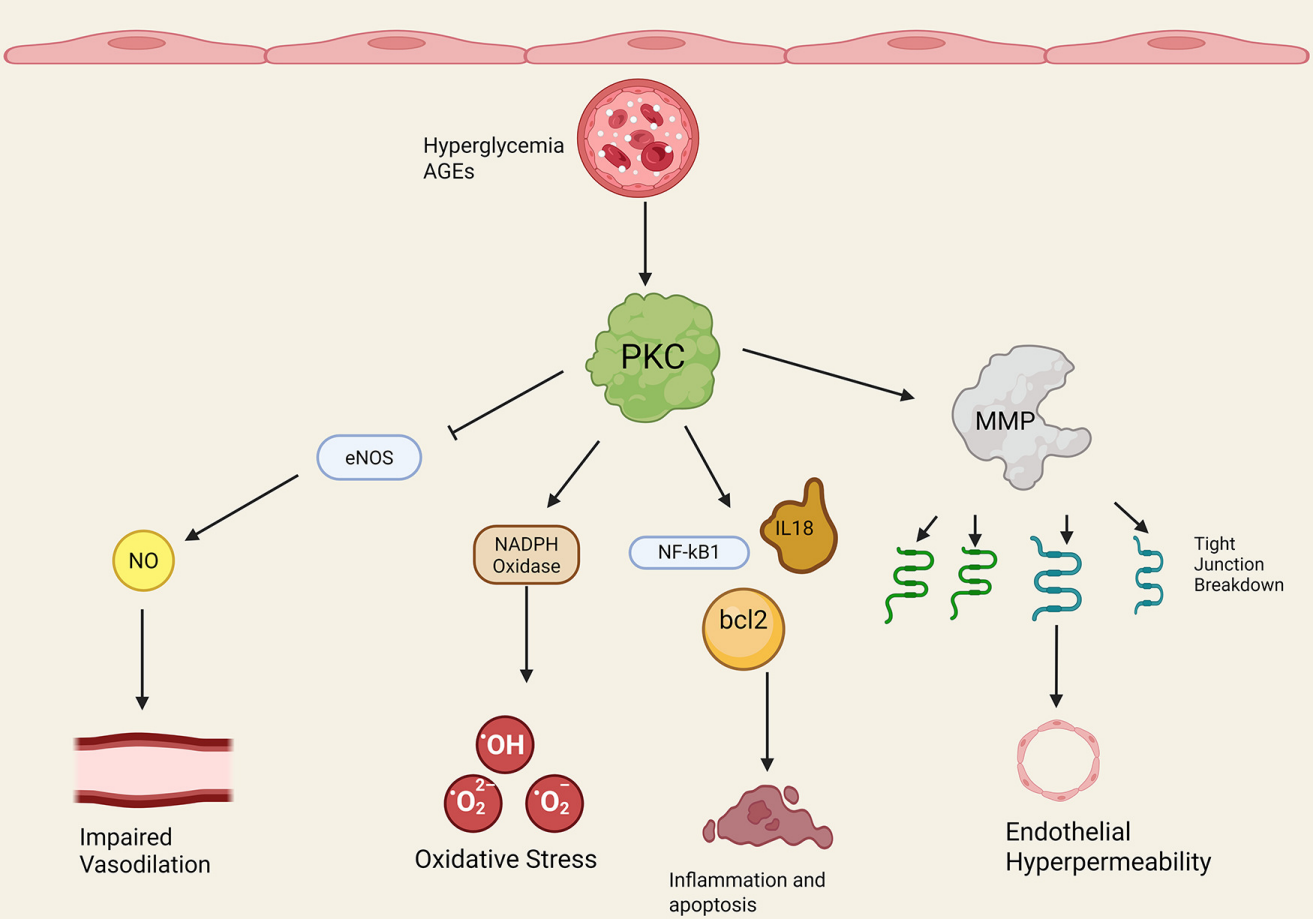
PKC and diabetic endothelial dysfunction. Note that straight arrowheads indicate activation and blunt arrows indicate inhibitory activity. Hyperglycemia and advanced glycosylation end products (AGEs) increase PKC activation/expression. PKC then has a number of downstream effects. PKC blocks endothelial nitric oxide synthase (eNOS) activity, leading to decreased nitric oxide (NO) and decreased endothelium-dependent vasodilation in the microvasculature. PKC increases endothelial NADPH oxidase activity through increased p47phox subunit translocation, leading to increased ROS production and oxidative stress. PKC promotes endothelial transcription of NF-kB, bcl2, IL-18, and other inflammatory and cell cycle proteins that derange the cell cycle and promote inflammation. Finally, PKC promotes matrix metalloprotein activity (MMP) and endothelial tight junction breakdown in diabetes, leading to increased endothelial permeability. Created in BioRender. Kant, S. (2025) https://BioRender.com/ogf60xc.

Figure 2 provides an overview of PKC and diabetic endothelial dysfunction (Figure 2). A variety of possible mechanisms have been investigated to elucidate the role of PKC in diabetic microvascular dysfunction. One mechanism centers on the pivotal role of endothelial nitric oxide synthase (eNOS), an enzyme responsible for the production of nitric oxide (NO), also known as endothelium-derived hyperpolarizing factor and a key mediator of endothelium-dependent vasodilation. Hypo and hyperglycemia increased PKC levels in bovine aortic endothelial cells exposed to low, normal, and high glucose media (38). Cells exposed to high glucose media exhibited impaired actin cytoskeletal alignment and NO release in response to simulated shear stress, reflecting a maladaptive response that increases vulnerability to atherosclerosis (38).

Lu et al. incubated human aortic endothelial cells with high glucose, and found that hyperglycemic conditions markedly increased PKCβ levels while decreasing eNOS levels (39). Selective inhibition of PKCβ prevented this; in addition, a transgenic calpain-1 knockout mouse model showed similar results, by blocking increased PKC activity and decreased eNOS activity (39). Calpain-1 is known to play a role in cytoskeletal protein remodeling, ATP synthase disruption, and regulation of MPTP opening in mitochondria; this study suggests that calpain-1 might also regulate a dysfunctional PKC-eNOS pathway in diabetic microvascular disease. Other studies have also confirmed a role for PKCβ inhibition in augmenting eNOS expression and increasing nitrite production in human aortic endothelial cells incubated in hyperglycemic conditions (40).

Next, human superficial forearm vein endothelial cells taken from diabetic patients exhibited increased eNOS phosphorylation without a change in total eNOS expression, and increased PKCβ expression (41). Diabetic patients in this study also exhibited lower brachial artery flow mediated dilation (measured by high resolution ultrasound measurements of brachial artery diameter before and 1 min after 5 min cuff occlusion of the upper arm), consistent with impaired vascular tone (41). Finally, PKCβ inhibition reduced basal eNOS activity and improved insulin-mediated eNOS activation in diabetic microvascular endothelial cells, suggesting a link between PKCβ activity, impaired eNOS activity, diminished NO production, and poor vasomotor tone.

### 3.4 PKC, ROS, and cell cycle dysregulation in the diabetic vascular endothelium

An explosion of recent studies provides strong evidence that PKC interferes with reactive oxygen species (ROS) homeostasis and promotes cell cycle dysfunction in diabetic microvascular endothelial cells. Advanced glycosylated end products (AGEs) are a family of compounds formed by non-enzymatic reactions between reducing sugars and proteins, lipids, or nucleic acids that have increasingly been identified as key mediators of cellular toxicity in hyperglycemic states, and may be one factor facilitating these interactions. Indeed, in a mouse model, AGEs facilitated proliferation of cardiac microvascular endothelial cells, increased phosphorylation of the extracellular signal related kinase (p-ERK), and stimulated entry of cells from G1 to S + G2/M phase of the cell cycle (42). AGEs also increased expression of survivin and decreased expression of cleaved caspase 3, tipping the balance of proliferation vs. apoptosis in favor of the former. Importantly, AGEs upregulated phosphorylation of PKC β1 and 2 in mouse cardiac microvascular endothelial cels, and inhibition of PKC β1 and 2 reversed all of the pro-proliferative effects detailed above (42).

A number of studies more directly studying hyperglycemia and apoptosis suggest that unlike the reported anti-apoptotic effects of AGEs reported by Luan et al., hyperglycemia may actually promote a more pro-apoptotic cellular microenvironment (42–44). For example, PKCβ inhibition in diabetic human coronary arteriolar endothelial cells increased the levels of the anti-apoptotic mediator p-Akt (45). Moreover, PKCβ inhibition did not affect expression levels of other critical cell survival signals such as bcl-2, catalase, superoxide dismutase type-1, or even eNOS (45). Meanwhile, Qin et al. found that PKCβ inhibition with apigenin and naringenin did upregulate bcl-2 expression and Akt phosphorylation in human umbilical vein endothelial cells exposed to high glucose conditions; the authors also observed decreased bax and caspase 3 activity, along with decreased ROS production (46).

Other studies investigating PKC inhibition and ROS production in human umbilical vein endothelial cells exposed to high glucose media have confirmed a link between heightened PKC β activity and increased NADPH oxidase activity, leading to increased oxidative and nitrosative stress markers and apoptosis (47–49). Wang et al. showed that metformin, PKCβ1 inhibition, and paeoniflorin all mitigated oxidative stress, inflammation, and apoptosis in human umbilical vein endothelial cells exposed to intermittent hyperglycemia (44). Ihnat et al. took this one step further, finding that human umbilical vein endothelial cells exposed to 2 weeks of continuous high glucose still exhibited elevated levels of PKCβ, NADPH oxidase subunit p47phox, bcl-2 associated protein X, and 3-nitrotyrosine 1 week after complete normalization of glucose levels (50). Pretreatment with antioxidants (such as alphalipoic acid, oxypurinol, or apocynin) prevented this sustained induction response (50). Findings such as this lend credence to a model of “cellular memory of high glucose stress” that causes persistently high ROS levels and endothelial dysfunction even after normalization of extracellular glucose levels (50).

Regulation of the NF-κβ signaling cascade has emerged as another means by which PKCβ in particular may influence the cell cycle in endothelial cells. To start, high glucose stress induced sustained PKCβ activation, and nuclear translocation of NF-κβ, in human umbilical vein endothelial cells (51). Those cells in turn experienced cell cycle arrest in G0/G1 phase, and displayed a tendency towards increased early apoptosis. MAPK3 expression was also increased in hyperglycemic human umbilical vein endothelial cells alongside PKCβ2 (51). This raises the possibility that MAPK3 may activate PKC via PLA2 group IVA; activated PKC in turn may further stimulate MAPK3, creating a self-perpetuating feedback loop (52, 53). Alleboina et al. found that hyperglycemia activated NF-κβ pathways in an Ins2Akita T1DM mouse model (54). Prolonged hyperglycemia impaired ischemia-induced activation of the canonical NF-κβ pathway in their mouse model, evidenced by impaired phosphorylation and degradation of IkB-α, through activation of PKCβ; PKCβ inhibition restored this (54).

### 3.5 PKC and endothelial barrier integrity in the diabetic vasculature

An assortment of studies show that increased PKC activity promotes disruption of endothelial adherens and tight junctions, compromising endothelial barrier integrity and promoting increased paracellular permeability and endothelial injury. Indeed, human umbilical vein endothelial cells treated with high glucose concentrations show increased VE-cadherin tyrosine phosphorylation, increased dissociation of VE cadherin-β catenin complexes, and increased transendothelial monocyte migration, all indicators of abnormal barrier function (55). These endothelial cells also showed increased activation of endothelial PKC and increased PKC-mediated ERK and myosin light chain phosphorylation. Likewise, an *in vitro* blood brain barrier (BBB) model using human brain microvascular endothelial cells exposed to hyperglycemia showed enhanced total PKC activity, driven primarily by PKCβ, increased MMP2 activity, and decreased occludin levels, again pointing to compromised barrier integrity (56).

Gaudreault et al. tested coronary microvascular permeability in a Zucker-DM diabetic rat model, and found increased PKCβ protein expression and decreased PKCδ expression in mouse coronary artery endothelial cells (57). In this same study, human coronary microvascular endothelial monolayers engineered to overexpress PKCβ or underexpress PKCδ exhibited impaired cell-cell adhesive barrier integrity, which raises an interesting notion of counter-regulatory effects (57). Perhaps PKCβ and PKCδ oppose each other's effects on coronary endothelial barrier integrity, creating a homeostatic balance that is disrupted in conditions such as diabetes.

With respect to PKCβ specifically, an assortment of studies using animal models and *in vitro* human endothelial cells taken from a variety of vascular beds show that inhibition of PKCβ protects microvascular barrier and monolayer barrier functions and decreases trans-endothelial migration (56, 58–61). Here, it is also important to highlight Dang et al., who tested both PKCβ and PKCα inhibition, and surprisingly found that PKCα inhibition with hypocrellin A had an even more pronounced effect on attenuating hyperglycemia-induced endothelial hyperpermeability in their human umbilical vein endothelial cell model than PKCβ inhibition (61). Therefore, while the bulk of current literature has focused on PKCβ in hyperglycemia-related endothelial barrier disruption, further research is needed to clarify a potential role of PKCα in this pathology as well.

### 3.6 PKC, endothelial inflammation, and atherogenesis in the diabetic vasculature

PKC promotes action of inflammatory cytokines and upregulation of endothelial cell adhesion molecules, both of which contribute to a pro-inflammatory and atherogenic microenvironment in diabetic vasculature. First, STZ-DM mice fed a western diet showed increased IL-18 and decreased IL-18 binding protein expression; PKCβ inhibition with ruboxistaurin (RBX) prevented both effects (62). High circulating IL-18 levels have been implicated in accelerated atherosclerosis and production of other inflammatory cytokines that promote plaque instability in diabetes. IL-18 also contributes to increased monocyte adhesion to endothelial cells, and promotes enhanced VCAM-1 expression. STZ-DM rats exposed to fluctuating hyperglycemia also exhibited marked elevations in TNF-α, PECAM1, and vWF; once again, PKCβ inhibition blunted all of these effects (44).

Of note, RBX treatment also reduced cholesterol ester levels in an STZ-DM mouse model and reduced macrophage levels compared to nontreated STZ-DM mice (62). Next, human aortic endothelial cells incubated with high glucose displayed increased VCAM-1 protein expression and increased membrane-bound PKCβ (63). PKCβ inhibition prevented VCAM-1 upregulation in a dose-dependent manner (63). Madonna et al. reported similar results in their own model of human aortic endothelial cells incubated under high glucose conditions (40).

### 3.7 PKC and endothelin-1 in the diabetic vasculature

Endothelin-1 is a potent vasoconstrictor and mediator of vasomotor tone, in particular through action on ETA receptors found on vascular smooth muscle tissue. Studies have shown that PKC may have a role in abnormal microvascular responses to endothelin-1 in diabetes; however, the specific role remains a matter of debate, as different studies point to conflicting roles for different PKC isoforms. For example, human chest wall skeletal muscle arterioles taken from diabetic patients exhibited decreased contractile responses to endothelin-1 (64–66). Application of the PKCα inhibitor salfingol further inhibited endothelin-1 mediated vasoconstriction (64–66). Meanwhile, primary human umbilical vein endothelial cells exposed to hyperglycemic conditions displayed increased endothelin-1 mRNA and protein expression, along with increased endothelin converting enzyme 1 protein (ECE1), PKCβ, and PKCδ isoform levels (67). Moreover, application of the PKCδ inhibitor rottlerin significantly reduced glucose-induced endothelin-1 secretion and ECE1 expression, while selective PKCα and β inhibitors had no effect. Clearly, further work is needed to clarify the roles of specific PKC isoforms in diabetic/hyperglycemic vasculature.

## 4 A deeper look: PKC and endothelial dysfunction in diabetic nephropathy

Diabetic nephropathy is a serious long term microvascular complication of T1 and T2 diabetes that occurs in 20%–40% of diabetic patients (68). Diabetic nephropathy presents with persistent albuminuria and a progressive decline in glomerular filtration rate (GFR), and is the leading cause of end stage renal disease (ESRD) in most of the developed world, including the United States (69). Diabetic nephropathy arises from hyperglycemic activation of several key molecular pathways involved in abnormal cell signaling, mesangial matrix proliferation, inflammatory cytokine and ROS generation, and glomerular basement membrane thickening (70–72). All of these eventually converge upon glomerular fibrosis, glomerulosclerosis, endothelial-mesenchymal transition, and ultimately, diabetic nephropathy. Given the significant morbidity and mortality burden imposed by diabetic nephropathy-related ESRD, it has become critical to establish a comprehensive understanding of the interconnected cellular and molecular pathways that foment diabetic nephropathy. Here, we highlight studies that implicated PKC as a potentially vital player in facilitating diabetic nephropathy.

On a basic level, PKC may directly blunt insulin signaling in diabetic glomeruli. Proof of this comes from observations that PKCβ inhibition with RBX enhances insulin action through phosphorylation of insulin- receptor substrate 1 (IRS1), Akt, eNOS, and GSK3α in STZ-DM and Zucker fatty insulin-resistant rats (73). Furthermore, RBX elevated total IRS1 levels in diabetic mouse glomerular endothelial cells (73). Next, there is evidence that PKC and VEGF act in concert to stimulate mesangial matrix proliferation in diabetic nephropathy. Hyperglycemia has been shown to stimulate VEGF expression, and VEGF in turn facilitates glomerulopathy and proteinuria in diabetic kidney disease (74). Using a rat model of cultured mesangial cells exposed to hyperglycemic growth conditions, Xia et al. demonstrated that inhibition of PKCβ1 and PKCζ blocked enhanced VEGF expression in hyperglycemic rat mesangial endothelial cells (74). Kelly et al. confirmed similar findings in an STZ-DM rat model, insofar as RBX attenuated increased VEGF mRNA production in glomerular endothelial cells and also reduced the extent of glomerular endothelial cell loss down to levels seen in nondiabetic mice (75).

PKC influence on NO production in the microvasculature at large in diabetes was discussed at length earlier; the same concept applies to diabetic nephropathy. Chu et al. took mouse kidney slices and exposed them to normoglycemic and hyperglycemic solutions, after which mouse glomerular capillary endothelial cells were examined for eNOS activity (76). Mouse glomerular capillary endothelial cells exposed to hyperglycemia exhibited a 60%–70% decrease in NO production relative to endothelial cells exposed to normoglycemic conditions (76). RBX prevented this, testifying to a role for PKCβ in depressing renal glomerular capillary eNOS activity.

Next, there is evidence that glycated albumin may directly stimulate glomerular PKC activity in diabetic nephropathy. Mouse glomerular endothelial cells incubated with glycated albumin displayed increased type IV collagen production (a critical component of the glomerular basement membrane), which was prevented by generalized PKC inhibition with GF109203X (77). Importantly, PKC inhibition blocked increased TFG β-1 mRNA and TGF β-1 protein production in glomerular endothelial cells (77). Similarly, Yasuzawa et al. showed that ethyl eicosapentaenoate inhibited PKCβ and TGFβ signaling in diabetic mouse kidneys, attenuating the extent of endothelial dysfunction and renal mesangial expansion (78). Such results suggest that glycated albumin activates PKC, which in turn stimulates glomerular basement membrane collagen deposition possibly through a TGF β-1 mediated mechanism. This would facilitate glomerular basement membrane hypertrophy, abnormal glomerular endothelial barrier integrity, and promote glomerular endothelial dysfunction.

Finally, PKCβ may block action of GLP1 in diabetic glomeruli, providing another means by which it exerts pathologic effects in the diabetic renal microvasculature. First, STZ-DM mice exhibited increased PKCβ activation and increased degradation of GLP-1 receptor (GLP-1R) via ubiquitination, enhanced phospho-c-Raf(ser338), and enhanced angiotensin II activation of phospho-Erk1/2 (79). Of note in this study, diabetic mice engineered to further overexpress PKCβ beyond the effects of hyperglycemia alone showed even greater losses of GLP-1R expression, heightened albuminuria, and profound mesangial matrix expansion beyond diabetic controls (79). Yin et al. explored whether supplemental GLP-1 treatment could mitigate against these effects, and found that glomerular microvascular endothelial cells of STZ-DM rats treated with recombinant human GLP1 had diminished PKCβ activity, increased PKA activity, and an overall reduced oxidative stress burden (80).

## 5 A deeper look: PKC and endothelial dysfunction in diabetic retinopathy

### 5.1 Overview

Diabetic retinopathy is another devastating microvascular complication of longstanding diabetes with a significant degree of morbidity. According to one study, an estimated 9.6 million people in the US, or about 26% of adults with diabetes (T1 and T2), had some degree of diabetic retinopathy (81). About 1.84 million people (or 5.06% of diabetics) had severe, vision threatening diabetic retinopathy (81). This represents a substantial increase in prevalence over the last decade. Runaway neovascularization and vascular hyperpermeability are hallmarks of endothelial dysfunction in diabetic eyes, leading to macular edema and proliferative diabetic retinopathy. A growing body of evidence suggests that PKC impacts retinal endothelial dysfunction and proliferation, making a contribution to the development of diabetic retinopathy (82). For example, Shiba et al. found increased DAG levels and PKC activity in STZ and BB diabetic rat endothelial cells, and Knott et al. found that PKC inhibition reversed the decline in DNA synthesis in bovine retinal endothelial cells exposed to high glucose conditions (83, 84). DeBosch et al. found that IGF-1 stimulated increased PKC activity and PI3 kinase activity in bovine retinal endothelial cells, and inhibition of PKCβ interfered with IGF-1 mediated retinal glucose transport (85).

### 5.2 PKC and VEGF in diabetic retinal endothelial cells

A growing body of literature points to extensive cross-talk between PKC and VEGF in diabetic retinal endothelial cells, purporting that PKC activation may promote retinal microvascular angiogenesis and retinal neovascularization through a VEGF signaling pathway (86). We begin with a review of results from mouse models. A transgenic mouse model engineered to overexpress PKCβ2 exhibited significantly increased angiogenic responses to oxygen-induced ischemia in retinal endothelial cells (87). Similarly, Zhang et al. found that STZ-DM rat retinal tissue exhibited increased PKCβ2 and VEGF mRNA and total protein (88). Treatment with total lignans from Fructus Arctii (dried ripe fruit of Arctium lappa) decreased the extent of PKC and VEGF overexpression, and improved retinal lesions in histopathological and electron-microscopic examination of diabetic mouse retinal tissue (88). To directly test interrelationships between VEGF and PKC in retinal endothelial cells, Aiello et al. performed intravitreal injections of VEGF in a rat model (89). This resulted in profound activation of retinal PKC, and translocation of PKCα, β2, and δ to retinal endothelial cell membranes and significantly increased retinal vasopermeability (89).

Turning to a different set of models, VEGF-treated bovine retinal endothelial cell cultures exhibited significantly increased overall glucose uptake with an increase in plasma membrane glucose transporter GLUT-1, along with a 90% increase in PKC activity (90). Importantly, the increase in plasma membrane GLUT-1 was not accompanied by an increase in total GLUT1 mRNA, suggesting that VEGF promoted translocation from intracellular stores to plasma membrane. PKCβ inhibition and cellular PKC depletion blocked VEGF-stimulated GLUT1 translocation (90). Next, treatment of bovine retinal endothelial cells with AGEs significantly increased VEGF expression, PKCβ translocation, ERK1 and 2 activity, NF-κβ activation, and endothelial cell proliferation (91). Treatment with gliclazide and vitamin E prevented AGE-induced pathology (91).

Li and Renier built on these results to show that bovine retinal endothelial cells incubated with AGEs showed increased ROS generation, increased VEGF expression and increased translocation of PKC β2 (92). Again, PKCβ inhibition appeared to have a protective effect against all of these AGE-induced changes. Finally, to more directly confirm the applicability of these findings to human tissue, human retinal vein endothelial cells cultured in high glucose media also exhibited increased VEGF-induced PKCβ2 translocation from cytosol to cell membrane, and an increased trend towards hyperproliferation (93).

### 5.3 PKC and blood-retinal barrier dysfunction in diabetic retina

PKC has been implicated in blood-retinal barrier (BRB) dysfunction in diabetic eye disease. First, at baseline, STZ-DM rats exhibit increased retinal expression of CRP, ICAM-1, and VCAM-1, along with decreased levels of occludin, claudin-5, and ZO1, key tight junction proteins (94). Together, these aberrations manifested as increased BRB permeability and retinal thickness. Application of endostatin, an endogenous suppressor of VEGF, reversed these changes, and also reversed upregulation of PKCβ and TGFβ in diabetic rat retinas (94).

With respect to PKC activity specifically, another STZ-DM rat model displayed a 71% increase in membrane specific PKC activity in retinal endothelial cells relative to controls (95). In this same study, rat retinal endothelial cells exposed to hyperglycemic conditions showed increased total DAG when compared with controls, with DAG being a known driver of PKC activity (95). Likewise, PKCβ has been shown to directly target phosphorylation of serine 490 of occludin, promoting ubiquitination and degradation of tight junctions in bovine retinal endothelial cells (96). To more closely examine this, Murakami et al. performed a site-directed mutagenesis of serine 490 to alanine 490, and discovered that this mutation blocked PKCβ mediated retinal microvascular hyperpermeability (96).

Another component of BRB dysfunction in diabetic retinopathy is abnormal endothelial cell-leukocyte adhesion; PKC appears to have a hand in this. First, studies show that leukocytes taken from blood samples of patients with T1 and T2 diabetes showed increased adhesion to bovine retinal capillary endothelial cells, and increased activity of core 2 glycosylating enzyme β1, 6 acetylglusoaminyltransferase (core 2 GlcNAc-T) (97, 98). The latter point becomes relevant because *in vitro* data suggests that hyperglycemia stimulates PKCβ-dependent phosphorylation of core 2 GlcNAc-T to promote increased leukocyte adhesion to bovine retinal capillary endothelial cells, with PKCβ inhibition attenuating this process (97). TNF-α has also been shown to act in concert with PKCβ2 phosphorylation to promote GlcNAc-T mediated increased leukocyte adhesion and capillary occlusion in diabetic retinopathy (98).

### 5.4 PKC and endothelin-1 in diabetic retinal endothelial cells

**Figure 3:**
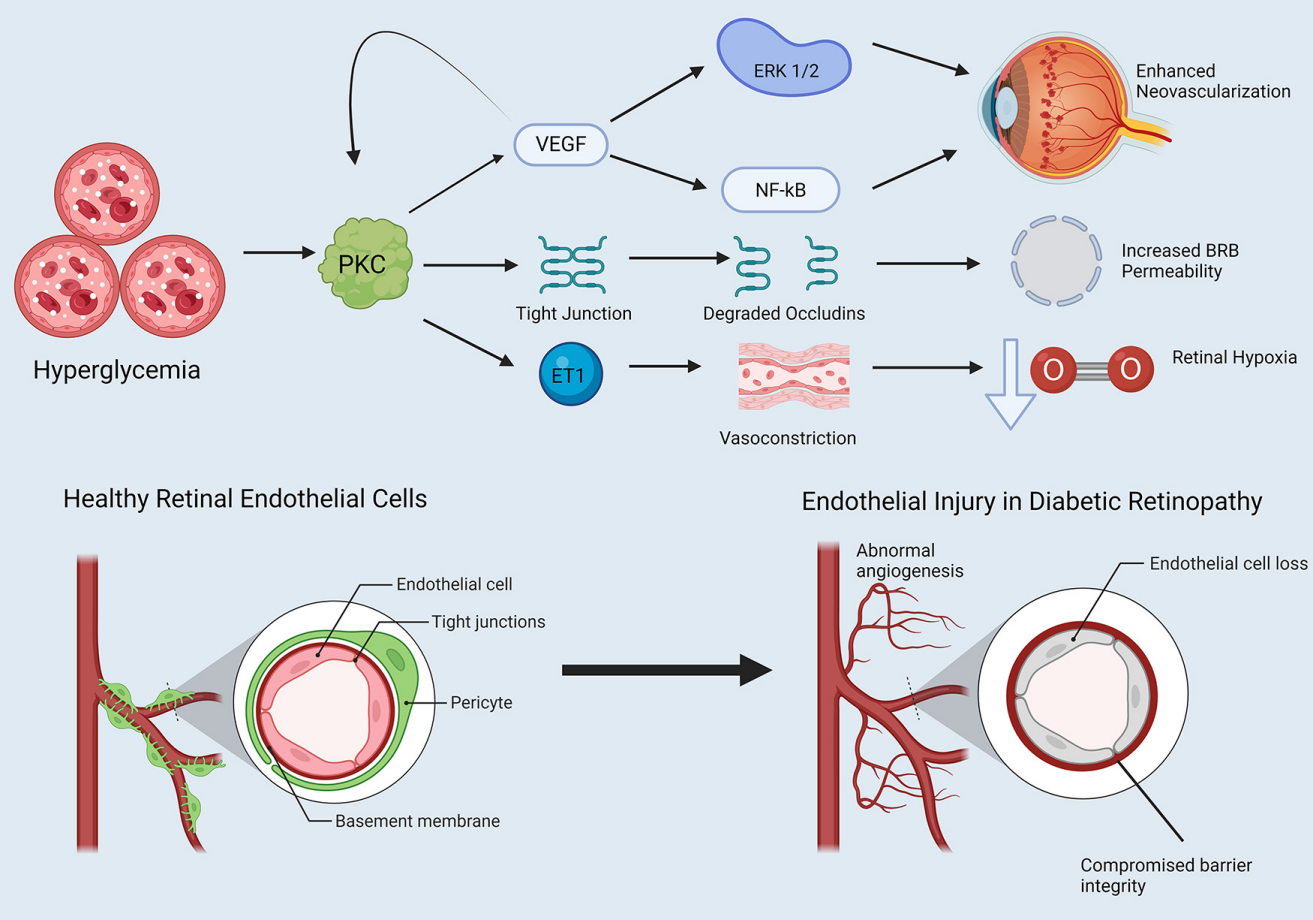
PKC and endothelial dysfunction in diabetic retinopathy. Note that straight arrowheads indicate activation and blunt arrows indicate inhibitory activity. Hyperglycemia influences PKC activation, which has several downstream effects. PKC induces VEGF expression, which itself has a positive feedback loop further activating PKC. VEGF promotes activation of proteins such as ERK 1 and 2 and NF-kB, which alters the cell cycle in retinal endothelial cells in favor of neovascularization. PKC degrades retinal endothelial tight junctions, increasing blood-retinal barrier (BRB) permeability. Finally, PKC enhances endothelin-1 activity in retinal endothelial cells, promoting retinal vasoconstriction and retinal hypoxia. Created in BioRender. Kant, S. (2025) https://BioRender.com/gvphk9g.

Enhanced endothelin-1 activity has been associated with decreased retinal blood flow in diabetic animals, and hyperglycemia may increase retinal endothelin-1 secretion. PKCβ may promote induction of endothelin-1 in the diabetic retina. Here, we turn to an STZ-DM mouse model and bovine retinal capillary pericytes exposed to high glucose, both of which showed increased prepro-endothelin-1 mRNA levels and increased PDGF-β (99). Application of PKC inhibitors blocked PKC-mediated endothelin-1 mRNA upregulation in both models (99). Park et al. observed similar results, finding that bovine retinal capillary endothelial cells and pericytes exposed to high glucose for 3 days exhibited 2-fold increases in endothelin-1 mRNA levels, along with increased membrane PKC activity (100). PKCβ inhibition prevented these changes (100). Finally, immunostaining showed that PKCβ2 and δ isoforms were significantly increased in bovine retinal capillary endothelial cells exposed to hyperglycemic conditions relative to other isoforms (100). Figure 3 summaries PKC effects in promoting vascular dysfunction leading to diabetic retinopathy (Figure 3).

## 6 PKC and endothelial dysfunction in hypoxia/ischemia-reperfusion

### 6.1 Background

Aerobic metabolism sustains all organ systems in the human body; disruption of oxygen homeostasis significantly impairs the function of all major organ systems. The degree of disruption varies, with certain organs (e.g., skeletal muscle) displaying more resistance to intermittent/brief hypoxia than others (e.g., the cardiopulmonary systems and central nervous system/CNS), which are exquisitely vulnerable to mere minutes of hypoxia/ischemia. Microvascular disease is a significant contributor to ischemic pathology in humans, manifesting as transient and chronic microvascular occlusion due to atherosclerosis, thrombosis, or disrupted microvascular vasomotor and myogenic tone. Ischemia may also occur in controlled medical settings, such as cardiac surgery involving cardiopulmonary bypass (CP/CPB), where a heart-lung machine temporarily assumes respiratory and circulatory function to facilitate open heart surgery. Of note, although many advances have been made in cardioprotective strategies over the years, ischemia-reperfusion injury, most commonly seen postoperatively as coronary endothelial dysfunction and a propensity towards coronary vasospasm and poor myocardial perfusion, remains a significant problem imposing substantial morbidity and mortality on patients (101, 102).

At the cellular level, ischemia disturbs cellular metabolism on a number of fronts. First, oxygen deprivation forces a shift towards anaerobic respiration, which elevates intracellular lactate. Lactate lowers intracellular pH, compromising critical maintenance enzymes that require a tightly regulated optimal pH range to function. Moreover, ischemia leads to heightened cell membrane permeability to calcium and promotes release of calcium from intracellular stores. Calcium in turn activates protases, nucleases, and phospholipases that degrade cytoskeletal proteins, DNA, and plasma membrane phospholipids. The end result is a tendency towards increased free radical production, diminished ROS clearance, mitochondrial disruption, and cell cycle dysregulation that will inexorably move towards apoptosis or necrosis in the absence of intervention (103, 104).

Unfortunately, even though the only treatment for ischemia is restoration of adequate oxygenation, reperfusion comes with its own set of short-term adverse effects (105–107). A rapid increase in oxygen in hypoxic/ischemic tissue may trigger oxygen overload that temporarily increases ROS production in an environment already extremely vulnerable to free radical/ROS/reactive nitrogen species (RNS) mediated injury. ROS may also cause further calcium overload even as hypoxic cells already suffer from excessive intracellular calcium activity. This combination of effects explains the transient increase in apoptosis often observed histologically in reperfused tissue (e.g., reperfused myocardial tissue).

Given the significance of ischemia-reperfusion injury as a major driver of pathology in a variety of disease states, we will now consider the role of PKC in mediating endothelial dysfunction in hypoxia/ischemia reperfusion.

### 6.2 PKC and vasomotor tone in hypoxia/ischemia-reperfusion

**Figure 4:**
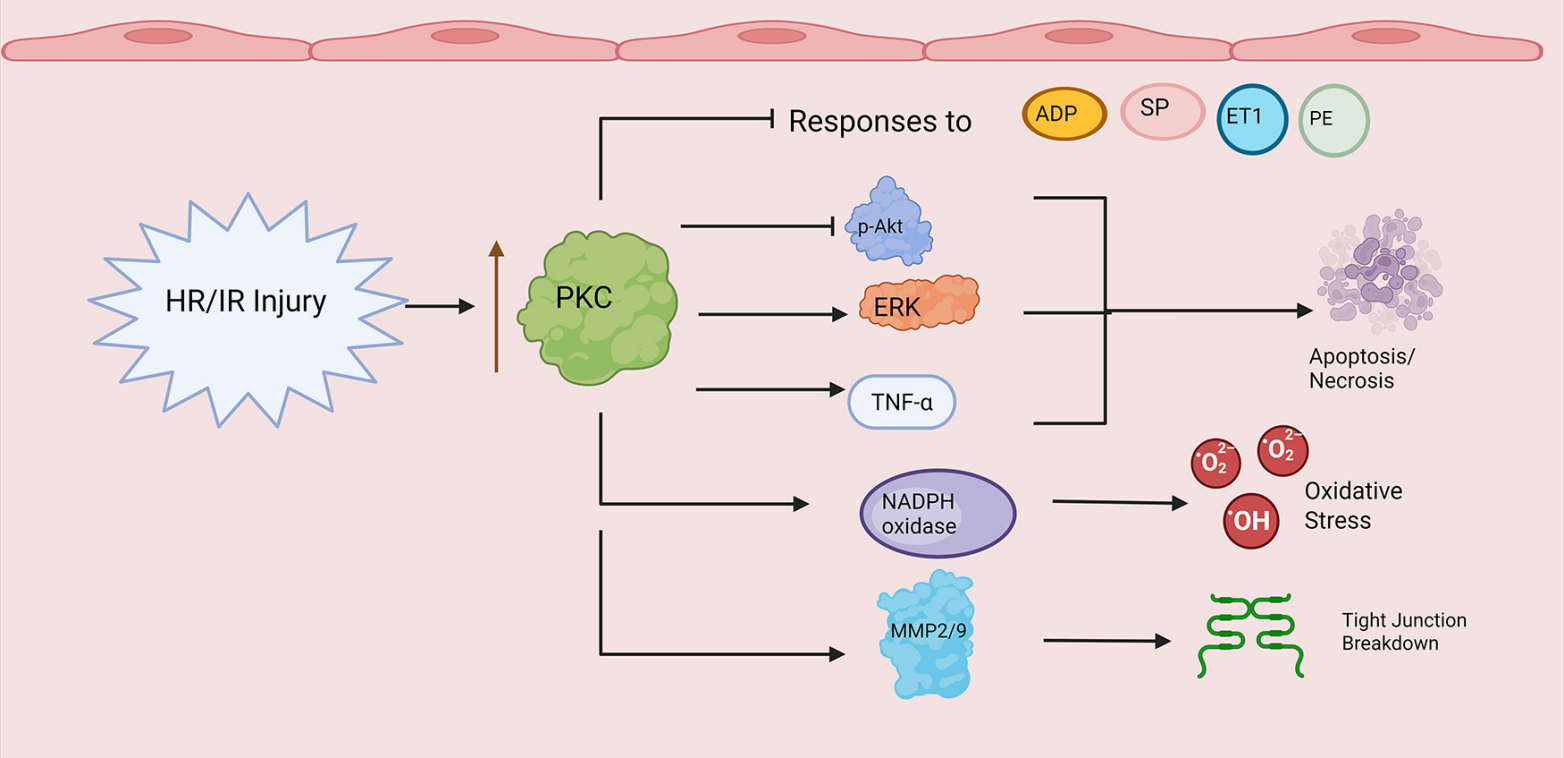
PKC and endothelial dysfunction in hypoxia/ischemia-reperfusion. Note that straight arrowheads indicate activation and blunt arrows indicate inhibitory activity. Hypoxia/Ischemia Reperfusion Injury (HR/IR) enhances PKC activity/expression. PKC promotes decreased endothelial responses to ADP, substance P (SP), endothelin-1 (ET1), and phenylephrine (PE). PKC alters expression levels of cell cycle proteins (e.g., p-Akt, ERK) and inflammatory mediators (e.g., TNF alpha) leading to apoptosis and necrosis. Finally, PKC increases NADPH oxidase and matrix metalloproteinase activity during HR/IR, leading to enhanced reactive oxygen species production (ROS) and compromised endothelial barrier integrity through tight junction breakdown. Created in BioRender. Kant, S. (2025) https://BioRender.com/b5h1lfx.

Figure 4 provides an overview of PKC activity in vascular endothelial hypoxia/ischemia-reperfusion injury. First, a growing body of literature provides evidence showing that PKC plays a role influencing abnormal vasomotor tone in hypoxia/ischemia-reperfusion. We begin with the endothelium-dependent vasodilators adenosine diphosphate (ADP) and substance P (SP). Here, both mouse coronary arterioles subjected to hyperkalemic cardioplegic hypoxia/reoxygenation and human skeletal muscle arterioles harvested before and after CPB exhibited decreased responses to ADP and SP post-treatment/procedure (37, 108). Treatment with the PKCβ inhibitor RBX conferred a protective benefit on coronary microvascular tone, promoting recovery of coronary relaxation responses to ADP and NS309 mediated potassium currents (108). However, while PKCβ overactivity appears to impair endothelium-dependent vasodilation, activity of other PKC isoforms may have counter-regulatory effects. For example, rat aortic segments exposed to PKCε activators exhibited increased endothelial NO release in a rodent model of ischemia-reperfusion injury relative to untreated controls (109).

Similarly, Shi et al. found that the compound hyperoside may protect against ischemia-reperfusion induced injury in rat basilar artery endothelial cells by enhancing overall PKC expression and inducing transient receptor potential vanilloid-type 4 channel (TRPV4)-mediated vasodilation (110). Unfortunately, this study did not subtype PKC isoforms to assess differential expression. Regardless, such conflicting findings raise the possibility that different PKC isoforms, and the overall balance of said isoforms, may vary from microcirculation to microcirculation, contributing to different effects on endothelium-dependent vasodilation in hypoxia/ischemia-reperfusion.

Turning to other vasoactive mediators, human skeletal muscle arterioles taken from patients before and after CP/CPB showed diminished phenylephrine-induced vasoconstriction following CPB (111). This effect was blunted with PKCα activation and enhanced with PKCα inhibition; moreover, overall PKCα expression decreased in coronary and skeletal microvessels following CPB (111). Meanwhile, a guinea pig model of global ischemia/reperfusion showed impaired coronary microvascular responses to acetylcholine following ischemia/reperfusion (112). This effect could be prevented by pretreating with the PKC inhibitor chelerythrine (112).

Endothelin-1 is another critical vasoconstrictor that has been implicated in vasomotor dysfunction following ischemia-reperfusion injury, coronary vasospasm, and cardiac allograft vasculopathy (113). Indeed, human saphenous vein endothelial cells incubated with high levels of endothelin-1 displayed decreased NO production and increased PKCδ and α translocation; however, overall PKC activity was significantly reduced compared with control cells (111). Moving to ischemic pathology, human skeletal muscle and coronary arterioles displayed significantly diminished contractile responses to endothelin-1 following CPB (114, 115). In both studies, the authors did not find a significant difference in endothelin receptor levels or PKCα levels after CPB (114, 115). Nonetheless, pretreatment of microvessels with the PKCα inhibitor salfingol protected against impaired endothelin-1 mediated contraction. Here, it is also worthwhile to note that the PKCα expression results reported in the above two studies contradict the results of Sodha et al., who studied the same microvascular systems and noted decreased PKCα expression levels (111). This discrepancy must be resolved to glean proper insight into the mechanisms underpinning what is clearly enhanced PKCα activity promoting impaired vasoconstrictive responses following ischemia-reperfusion injury.

### 6.3 PKC and endothelial cell cycle dysregulation in hypoxia/ischemia-reperfusion

A tendency towards enhanced apoptosis is a hallmark of hypoxia-ischemia reperfusion injury. First, results from animal models. Wei et al. found that RBX treatment guarded against apoptosis in mouse cardiac microvessels subjected to ischemia-reperfusion injury (58, 59). Likewise, Tang et al. observed that oxygen-glucose deprivation/reoxygenation (OGD/R) increased apoptosis in rat brain microvascular endothelial cells, and increased mRNA expression levels of PKCδ, Myristoylated Alanine-Rich C-kinase Substrate (MARCKS), and matrix metalloproteinase 9 (MMP9) (116). Treatment with the PKC inhibitor rottlerin increased rat brain microvascular endothelial cell viability while decreasing PKCδ, MMP9, and MARCKS mRNA expression (116). Next, PKCβ inhibition abolished VEGF upregulation of A-disintegrin and metalloproteinase with thrombospondin motifs 1 (ADAMTS1) to promote endothelial proliferation and angiogenesis in a mouse model of ischemia-induced retinal neovascularization (117).

Meanwhile, Bright et al. report that intraperitoneal injection of the PKCδ inhibitor δ-V1-1-TAT increased the number of patient microvessels and augmented overall cerebral blood flow in a rat model of acute focal ischemia induced by MCA occlusion (118). Finally, Kim et al. showed that bovine pulmonary artery endothelial cells exposed to OGD/R had increased LDH release and annexin V positive staining cells, both of which are hallmarks of apoptosis (119). Curiously, isoflurane treatment in the early phase of simulated reperfusion, along with application of the mitochondrial K(ATP) channel activator diazoxide, reduced the extent of endothelial injury; however, generalized PKC inhibition abolished both protective effects, pointing to a central role for PKC in a final common pathway leading to apoptosis during hypoxia/ischemia-reperfusion (119).

Turning to human tissue, Nho et al. found that human coronary arteriolar endothelial cells subjected to hypoxia/reoxygenation showed decreased levels of anti-apoptotic proteins such as p-Akt, with PKCβ inhibition protecting against this effect (45). Of note, these effects were even more pronounced in diabetic vessels made to undergo hypoxia/reoxygenation, likely due to compounding effects of factors discussed earlier in the diabetes section (45).

To further investigate the role of PKC, Shou et al. tested the effects of PKCδ silencing RNA treatment, and found that PKC delta silencing RNA increased cell viability in their hypoxia/deoxygenation injury model (120). Moreover, treatment with the PKC activator PEP005 augmented the deleterious effects of hypoxia/reoxygenation injury (120). Finally, TNF-α appears to enhance reoxygenation-mediated apoptosis in human coronary artery endothelial cells, with TNF-α being one of many pro-inflammatory cytokines released during hypoxia/ischemia as well (see below) (121). Of note, TNF-α-induced apoptosis could be mitigated by pretreatment with a generalized PKC inhibitor, suggesting that PKC is an effector by which TNF-α promotes apoptosis in reperfusion injury (121).

### 6.4 Endothelial inflammation, oxidative stress, and PKC in hypoxia/ischemia-reperfusion

Inflammation and oxidative stress are closely linked with cell death via necrosis and apoptosis. Heightened PKC activity in hypoxia/ischemia-reperfusion appears to promote inflammation and oxidative stress at the subcellular level. Consider first the effects of key inflammatory cytokines. Indeed, ischemia-reperfusion treatment in an *in vivo* rat model augmented coronary arteriolar TNF-α release, and subsequently, PKC and myosin light chain kinase (MLCK)-dependent protein extravasation in the rat coronary circulation (122). The same authors found separately that treatment of rat heart microvascular endothelial cells with TNF-α, IL-1β, and IL-6 all significantly increased PKC activation and endothelial monolayer hyperpermeability (122).

Moreover, human umbilical vein endothelial cells exposed to medium from hypoxic cardiac myocytes (exposed to <1% oxygen) showed increased COX2 mRNA expression and increased VEGF production, with selective PKCα and β inhibition attenuating these changes (123). The relationship between PKC and VEGF in mediating microvascular dysfunction was discussed at length before (see DM section). COX2 is a critical early actor in arachidonic acid metabolism and promotes production of prostaglandins such as PGE2 which promote macrophage activation, endothelial activation, and systemic inflammation.

A pro-inflammatory microenvironment created by cytokine storm during hypoxia-reoxygenation facilitates and acts in concert with increased ROS activity, with PKC acting as a putative intermediary. Mouse pulmonary microvascular endothelial cells in perfused lungs subjected to abrupt stoppage of blood flow exhibited increased ROS generation and increased activation of PI3K and Akt (124). PKC inhibition blocked ROS generation (124). Next, endothelial progenitor cells isolated from patients with stable coronary artery disease exhibited increased oxidative stress and poor *in vivo* angiogenesis capacity along with increased activation of NADPH oxidase, likely driven by increased membrane translocation of the NADPH oxidase activating subunit p47phox (125). Importantly, endothelial progenitor cells from coronary artery disease patients also displayed PKCα and β2 upregulation, with PKC inhibition reducing expression and activity of NADPH oxidase (125).

Granted, PKC does not influence all forms oxidative stress during ischemia/reperfusion; rather, the effects appear largely specific to its role in promoting NAPDH oxidase activity. For example, human umbilical vein endothelial cells exposed to ischemia/reperfusion have also been shown to exhibit increased hydrogen peroxide and mitochondrial ROS production (126). Administration of RBX or even a generalized PKC inhibitor did not have any effect on either, suggesting that while PKC inhibition can bring down oxidative stress through some pathways, it is not the be all-end all in preventing oxidative stress during hypoxia/ischemia-reperfusion.

### 6.5 PKC and endothelial barrier breakdown in hypoxia/ischemia-reperfusion

Through a combination of inflammation, oxidative stress, and direct activation of matrix metalloproteinases, PKC overactivity compromises endothelial and microvascular barrier integrity during hypoxia/ischemia-reoxygenation. A mouse-model of hindlimb ischemia demonstrated upregulation of matrix metalloproteinase (MMP) 2 and MMP9 in a PKC-mediated manner (127). PKC specifically increased MMP9 expression through activation of the ERK signaling pathway, and increased MMP2 expression through the p38, ERK, and c-Jun N-terminal kinase pathways (127). MMPs disrupt tight junctions by degrading occludin and claudin proteins, increasing intercellular permeability and contributing to tissue damage in a variety of disease states (128).

Kadir et al. created an *in vitro* human BBB model using co-culture of astrocytes and pericytes with human brain microvascular endothelial cells, and exposed this model to 4 h of OGD with and without PKCβ inhibitor treatment (129). They found that PKCβ inhibition protected BBB integrity, evidenced by increased transendothelial electrical resistance and decreased sodium fluorescin flux (129). This seems to have been accomplished through decreased ischemia-induced actin cytoskeleton remodeling, oxidative stress, and apoptosis in human brain microvascular endothelial cells (129).

Zhu et al. report similar results in their mouse brain microvascular endothelial cell BBB model exposed to OGD, noting increased BBB permeability, increased PKC levels, and decreased expression of tight junction proteins claudin-5 and ZO-1 (130). PKCβ inhibition also reduced ischemia-induced endothelial permeability in bovine aortic endothelial cells treated with KCN to inhibit oxidative phosphorylation and simulate ischemic conditions (131). Of note, in the aforementioned study, antisense oligodeoxyribonucleotides aimed at suppressing activity of specific PKC isoforms demonstrated that PKCα suppression almost completely inhibited ischemia-induced endothelial cell hyperpermeability in the authors' model system, while knockdown of PKCε and ζ had no effect (131).

## 7 PKC and endothelial dysfunction in hypertension

Hypertension is a leading cause of cardiovascular disease and premature death around the world. According to one study examining worldwide trends in hypertension, the number of people aged 30–79 with a diagnosis of hypertension (using criteria of systolic blood pressure 140 or greater, or diastolic blood pressure 90 or greater, or being on medication for hypertension) appears to have doubled between 1990 and 2019, from 331 million women and 317 million men to 626 million women and 652 million men (132). Turning to the US specifically, data from the National Health and Nutrition Examination Survey from 2017 to 2018 shows that 56.3% of US adults with hypertension have uncontrolled blood pressure, defined as SBP > 140 or DBP > 90 (133). Furthermore, hypertension in the US has been implicated as either a primary or major contributing factor in over half a million deaths (133).

Hypertension increases risk for a vast array of medical complications affecting all organ systems, including stroke, cardiomyopathy, renal dysfunction, retinopathy, and pulmonary hemorrhage (134). These all occur through a combination of macrovascular and microvascular complications. First, at the macrovascular level, essential hypertension causes arteriolosclerosis, characterized by myointimal cell hyperplasia and vascular smooth muscle proliferation (135, 136). This results in thickened, stiff media and large-sized arteries with reduced compliance, leading to impaired myogenic tone, and a vicious cycle of ever-worsening systemic blood pressures. Indeed, aortic endothelial cells taken from rats with two kidney-one clip (2K-1C) hypertension exhibited impaired endothelium-dependent (acetylcholine) and endothelium-independent (nitroglycerin) vasodilatory responses (137).

At the microvascular level, hypertension accelerates atherosclerosis and promotes endothelial injury (138, 139). Flow-mediated shear stress in the microcirculation increases endothelial permeability and platelet-endothelium interactions (138). In addition, hemodynamic stresses and turbulent flow have been connected to increased oxidative stress and excessive collagen deposition (140). The end result is significant pathological microvascular remodeling. For example, one study showed that coronary arterioles taken preoperatively from cardiac surgery patients with uncontrolled hypertension (defined by the authors as SBP > 130) exhibited markedly increased myogenic tone and enhanced epinephrine-mediated contractile responses compared to vessels taken from normotensive or controlled hypertensive patients (141).

**Figure 5:**
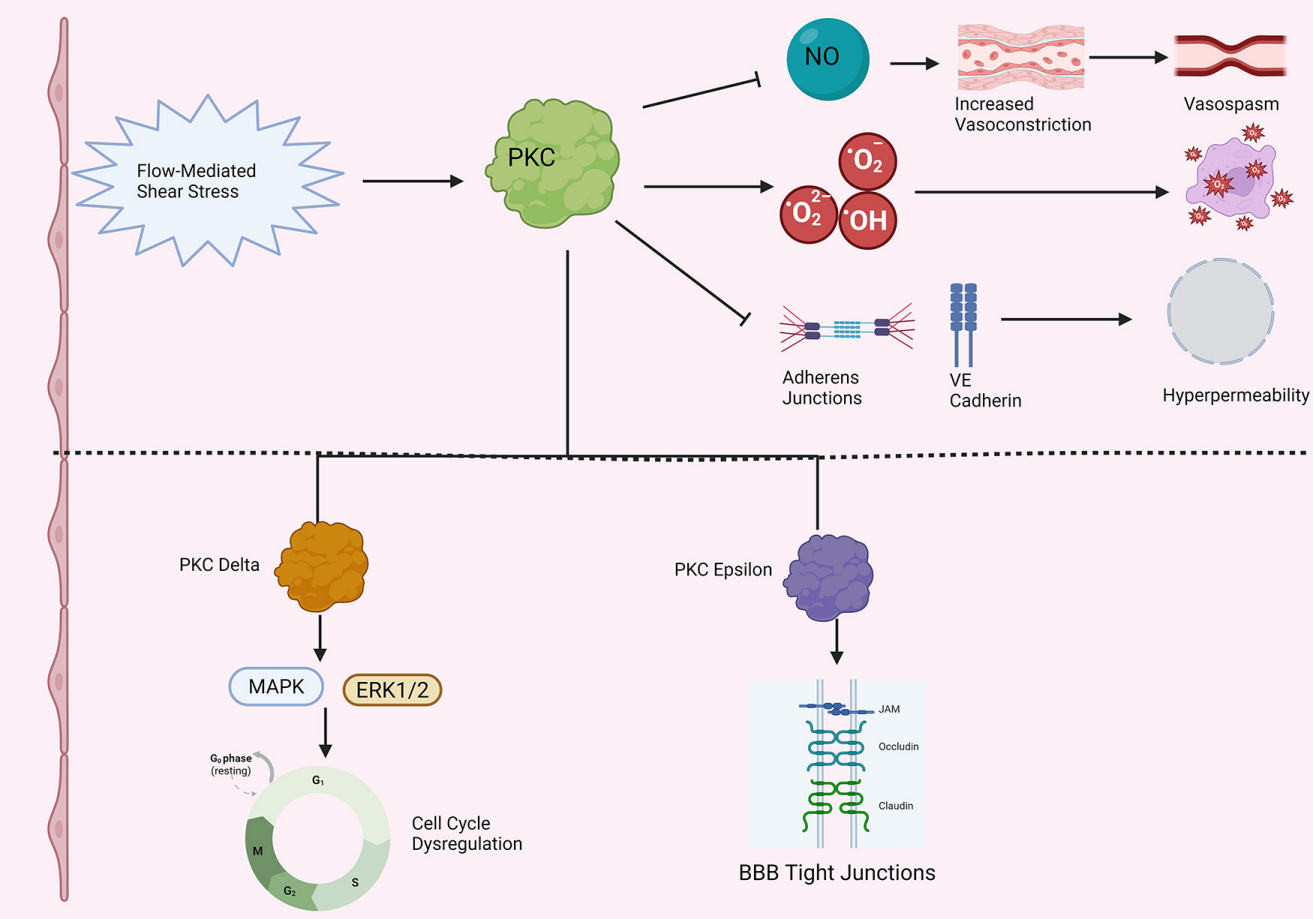
PKC and endothelial dysfunction in hypertension. Note that straight arrowheads indicate activation and blunt arrows indicate inhibitory activity. Flow mediated shear stress in hypertension activates PKC. (Top) Activated PKC decreases nitric oxide (NO) levels, leading to enhanced vasoconstriction, increased vasospasm, and increased propensity to arteriolar stiffness in the microvasculature. PKC increases ROS production, leading to oxidative stress and cell death. PKC also decreases adherens junction integrity and VE cadherin localization in the setting of hypertension, leading to increased microvascular endothelial permeability. (Bottom) More detailed breakdown of two specific PKC isoforms that have been more fully characterized in endothelial cells in the setting of hypertension. PKC Delta increases MAP kinase (MAPK) and ERK1/2 activity, dysregulating the cell cycle in endothelial cells. PKC epsilon overactivity has been associated with increased tight junction turnover in hypertensive endothelial cells. Created in BioRender. Kant, S. (2025) https://BioRender.com/ho8zltk.

PKC may have a role in hypertension-related endothelial dysfunction, although the literature on this subject is very much still emergent; refer to Figure 5 for an overview (Figure 5). First, porcine aortic endothelial cells cultured on stiff substrates to simulate increased arterial stiffness exhibited increased activity ROS and adherens junction loss in response to treatment with PMA, a potent activator of PKC (142). Importantly, treatment with ROS scavengers abolished the effects of PMA (142). Likewise, aortic endothelial cells from elastin haploinsufficient mice, which exhibit stiffer aortic endothelial cells than wild type mice, and had further decreased colocalization of the critical endothelial adhesion molecule VE cadherin following PMA treatment (142). Next, application of a generic PKC activator PDBu triggered increased vasoconstrictive responses in hypertensive rats vs. controls (137). Furthermore, conditioned medium of bovine aortic endothelial cells enhanced vascular smooth muscle Na/K pump activity to a far greater degree in spontaneously hypertensive rats than normotensive controls, with the effect reduced following treatment with PKC inhibitor staurosporine, pointing to a role for PKC in enhanced endothelium-dependent microvascular responses in hypertension (143). Finally, to add a therapeutic bent to the narrative, the widely used antihypertensive drug amlodipine may have a PKC-mediated effect. Indeed, He et al. show that amlodipine may increase flow-mediated dilation, NO, and eNOS activity in human umbilical vein endothelial cells through decreased PKC phosphorylation, and reduced PKC-dependent threonine 495 phosphorylation on eNOS (144).

The bulk of work investigating PKC and endothelial dysfunction in hypertension thus far has focused on two isoforms: PKCε and PKCδ. First, consider PKCδ. Intraperitoneal injection of PKCδ in Dahl hypertensive rats increased cerebral blood flow and reduced infarct size in stroke due to MCA occlusion (118). Furthermore, Dahl salt-sensitive hypertensive rats also exhibit significantly increased BBB permeability relative to control animals; treatment with a PKCδ inhibitor was shown to decrease BBB tight junction degradation in these hypertensive rats (145). Next, consider PKCε. Tseng et al. found increased levels of PKCε and ζ protein levels in bovine aortic endothelial cells exposed to flow-mediated shear stress, in conjunction with heightened activation off 42 kD and 44 kD MAP kinases (146). The former is consistent with data showing that blocking intracellular calcium mobilization with BAPTA did not affect increased PKC activity during flow stress, providing evidence that activity of either atypical or novel PKC isoforms drives these results (146). Likewise, antisense PKC-ε phosphorothioate oligonucleotides decreased PKCε levels by roughly 80% and completely inhibited shear stress-stimulated ERK 1/2 activation in bovine aortic endothelial cells (147). Such findings go hand in hand with the observation that shear stress activates PKCε, which activates Raf-1, itself an activator of ERK 1/2, through serine/threonine phosphorylation (148).

## 8 Clinical trials involving PKC inhibitors and future directions

Having completed an interrogation of studies examining PKC in the pathophysiology of diabetes, ischemia-reperfusion, and hypertension, we finish this review with a discussion of relevant clinical trials testing PKC inhibition in patients, and future avenues for investigation with an emphasis on clinical relevance.

### 8.1 Diabetes

Almost all clinical trials to date testing PKC inhibition in human subjects have been in the context of diabetes pathology. We begin with RBX and diabetic retinopathy. In a very small patient subset, Aiello et al. tested various doses of RBX (8 mg once daily, 16 mg once daily, and 16 mg twice daily) in a small group of 29 patients with T1 and T2 diabetes, and found that increasing doses of RBX up to 16 mg twice daily improved retinal circulation time and retinal blood flow, with abdominal pain as the only adverse effect (149). Turning to larger clinical trials, the Protein Kinase C β Inhibitor Diabetic Retinopathy Study (PKC-DRS) multicenter randomized clinical trial tested the effect of 32 mg/day RBX vs. placebo in 252 patients with nonproliferative diabetic retinopathy, and found that while RBX was well tolerated without significant adverse effects, RBX treatment had no effect on the primary endpoint of progression to proliferative DR; however, RBX treatment did show a clinical benefit in delaying time to occurrence of moderate vision loss (150). Likewise, the Protein Kinase C β Inhibitor-Diabetic Retinopathy Study 2 tested 32 mg/day RBX treatment in an even larger group of 685 patients, and found that sustained moderate vision loss occurred in 5.5% of the treatment group vs. 9.1% of the placebo group (151). RBX-treated patients also showed better mean visual acuity vs. placebo after 12 months of treatment, along with statistically significantly slower progression of macular edema (151, 152). After a two-year open label extension of this trial, RBX-treated patients continued to experience less sustained moderate visual loss than those in the placebo group (153).

Moving to diabetic nephropathy, a randomized, double-blind, placebo-controlled, multicenter, pilot study tested the effects of 32 mg/day RBX for 1 year in patients with T2 diabetes and persistent albuminuria [albumin-to-creatinine ratio (ACR) 200–2,000 mg/g], despite renin-angiotensin system (RAS) inhibitors (154). RBX treatment significantly reduced albuminuria and ACR after 1 year; in addition, the placebo group experienced a significant reduction in eGFR over the course of that year, while the treatment group did not (154). Unfortunately, between-group differences did not achieve statistical significance, which the authors attributed to the low power of the study. In another study of just over 100 patients with T2 diabetes and urinary ACR 200–2000 mg/g despite treatment with RAS inhibitors, RBX treatment produced a tendency to lower urinary TGF-beta to creatinine ratios (considered to be a marker of renal tubulointerstitial fibrongenesis) than placebo, although the difference did not achieve statistical significance (155).

With respect to diabetic neuropathy, a multinational, randomized, Phase II, double-blind, placebo-controlled parallel-group trial compared 32 mg/day or 64 mg/day RBX with placebo for 1 year, and tested measurable vibration detection (VDT) threshold as a proxy for extent of neuropathy (156). RBX treatment appeared to improve sensory symptoms and nerve fiber function compared to placebo, with the effect most pronounced in patients with clinically significant symptoms (156). Casellini et al. conducted a 6-month, randomized, double-masked, placebo-controlled study to evaluate the effects of RBX on skin microvascular blood flow, neurological deficits, sensory symptoms, and nerve conduction studies among other metrics of diabetic neuropathy, and found that RBX-treated patients had enhanced skin microvascular blood flow at the distal calf, reduced sensory symptoms, and improved overall quality of life metrics compared to placebo (157). Adverse effects were similar between groups; we will note that one patient in the RBX group experienced a myocardial infarction that resulted in death, and the investigators were not able to definitively rule out a casual link between RBX and myocardial ischemia (157).

Finally, Mehta et al. took a small group of patients and studied brachial artery flow mediated dilation, a surrogate for vascular function, in type 2 diabetic patients treated with RBX (158). 6 weeks of RBX treatment improved flow mediated dilation in diabetic patients relative to placebo (158). In a pilot study, Cherney et al. found similar results, showing that RBX treatment improved brachial artery flow mediated dilation in hyperglycemic conditions in type 1 diabetic patients (159). Such results suggest that RBX might have a protective effect on vascular function in general (outside of the specific contexts of retinopathy/nephropathy/neuropathy) in diabetes.

Despite all these promising clinical trial results, no large multi-center clinical trials testing RBX for diabetic vascular disease have occurred since the late 2000s/early 2010s. The last time the FDA weighed in on RBX as a potential drug was in the mid-2000s, when FDA advisors informed Eli Lilly that the pharmaceutical giant would need to run a new set of clinical trials in order to provide more data before approval of a New Drug Application. At that time, Lilly must have decided that the potential benefits of pursuing RBX further did not outweigh the costs of pushing new clinical trials. However, a great deal has changed over the last decade and a half; given the ever-advancing state of the literature and increasing evidence speaking to PKC as a major player in diabetes pathology, the time has never been more ripe to revisit the therapeutic potential of RBX (and other PKC inhibitors) in diabetic micro and macro vascular disease.

### 8.2 Ischemia-reperfusion and hypertension

Turning to ischemia-reperfusion, while there have been promising results in animal models and *in vitro* studies, there have been to date no major studies or clinical trials testing PKC inhibitors in mitigating ischemic microvascular pathology in human patients. For example, future studies can consider testing whether oral RBX, at doses shown to be potentially therapeutic for diabetic microvascular complications (e.g., 16 or 32 mg/day), can protect against postoperative coronary vasospasm and impaired vasomotor tone following cardiac surgery involving CP/CPB. Likewise, future investigators may consider examining the effects of RBX in humans post-revascularization/percutaneous coronary intervention during myocardial infarction, another important subdomain within ischemia-reperfusion injury—with a strong emphasis on assuring safety.

For hypertension, there is a dearth of studies involving human tissue generally, as the bulk of the literature thus far involves animal models and bovine aortic endothelial cells; this needs to be expanded upon to better clarify translation potential. In addition, given evidence discussed earlier that PKC activators augment vasoconstrictive responses in hypertensive animal models, and that amlodipine may somehow decrease PKC phosphorylation (which itself requires further investigation to directly elucidate potential mechanisms), there is a reasonable argument for testing PKC inhibitors for potentially augmenting effects of antihypertensive medications in humans (137, 144). One study from 2007 tested the potential of high dose RBX (96 mg/day) to augment the effects of nitroglycerin on systolic blood pressure in patients with chronic stable angina, only to find no significant effect of RBX (160).

### 8.3 PKC and endothelin-1

Finally, there are lingering general questions regarding the mechanism of action of PKC that will require further elucidation; chief among these is the murky connection between various PKC isoforms and endothelin-1. Indeed, while the literature is fairly conclusive on some aspects, such as PKC β promoting endothelin-1 activity in diabetic retinal endothelial cells, there are major discrepancies in other aspects (99, 100). In this review, we have discussed literature showing that while some studies show that PKC-α inhibitors attenuated already diminished endothelin-1-mediated responses in diabetic human skeletal micro vessels, other studies showed that PKC-α inhibitors had no effect on endothelin-1 activity in human umbilical vein endothelial cells (64, 65, 67). Perhaps there is an element of vessel/tissue bed specific effects at play in the diabetic microvasculature, and a more detailed interrogation of downstream elements of PKC-α signaling in these different types of vessels could provide answers. Furthermore, while some studies have shown no changes in PKC α expression levels alongside diminished contractile responses to endothelin-1 in human skeletal muscle arterioles following CPB, other studies in similar circumstances show decreased PKC α levels—and there is also evidence that PKC α inhibition protects against impaired endothelin-1 responses during ischemia-reperfusion (111, 114, 115). Perhaps postranslational modifications to PKC α enhance its ability to interfere with endothelin-1, or perhaps it is a matter of PKC α trafficking from membrane bound/inactive to cytosolic/active conformations without a change in actual protein expression levels. Both hypotheses merit further investigation in future studies.

## 9 Conclusion

PKC has an important role in vascular endothelial function. Multiple PKC isoforms are active in the vascular endothelium, with variable distributions of the various isoforms existing in the different microcirculations. Activation of PKC is a crucial step in intracellular signal transduction pathways that regulate virtually all aspects of cell proliferation, differentiation, and survival. PKC dysfunction has been implicated in microvascular endothelial dysfunction in disease states characterized by impaired metabolism, including diabetes, hypertension, and ischemia-reperfusion (see Table 1 for summary). Excessive PKC activity deranges the cell cycle, triggering abnormal angiogenesis in certain tissue beds (e.g., the diabetic retina or glomerular microvasculature) or augmenting apoptosis in other circumstances (e.g., in the aftermath of ischemia-reperfusion). PKC overactivity also disrupts ROS and RNS clearance, exacerbating oxidative stress, and creates pro-inflammatory microenvironments at the cellular and tissue levels. Finally, PKC hyperactivity compromises normal microvascular tone, and alters microvascular responsiveness to endothelium-dependent and independent vasodilators and vasoconstrictors. Future studies will need to expand on the current body of literature, which is largely based on *in vitro* human endothelial cell lines and animal models, and extend the findings discussed here to *in vivo* human studies. Ultimately, our hope is that improved understanding of PKC function and dysfunction will lead to novel targeted treatments aimed at protecting endothelial function across a variety of disease states.

**Table 1:** Vascular endothelial PKC activity and downstream effects in diabetes, hypoxia/ischemia-reperfusion, and hypertension.

Disease process	Vascular endothelial cell PKC activity	Downstream endothelial effects
Diabetes/Hyperglycemia (systemic circulation)	PKCα: Increased	NO Activity: Decreased
	PKCβ: Increased	NADPH Oxidase Activity: Increased
	PKCδ: Decreased	NF-κβ Pathway Activity: Increased
		Tight Junction Occludin Levels: Decreased
		MMP2 Activity: Increased
		TNF-α Activity: Increased
		IL-18 Levels: Increased
Hypoxia/Ischemia-Reperfusion	PKCα: Increased	Endothelium-Dependent Vasodilation: Decreased
	PKCβ: Increased	Phenylephrine Response: Decreased
	PKCδ: Increased	Acetylcholine Response: Decreased
		VEGF: Increased
		NADPH Oxidase Activity: Increased
		MMP2/9 Activity: Increased
		Claudin Levels: Decreased
		MARCKS, PI3K, Akt Expression: Increased
		TNF-α: Increased
Hypertension	PKCδ: Increased	Arterial Stiffness: Increased
	PKCε: Increased	VE Cadherin Colocalization: Decreased
	PKCζ: Increased	42 kD/44 kD MAP Kinase Activity: Increased
		ERK 1 and 2 Activity: Increased
